# Enhanced Room‐Temperature Photoluminescence Quantum Yield in Morphology Controlled J‐Aggregates

**DOI:** 10.1002/advs.201903080

**Published:** 2021-01-04

**Authors:** Surendra B. Anantharaman, Joachim Kohlbrecher, Gabriele Rainò, Sergii Yakunin, Thilo Stöferle, Jay Patel, Maksym Kovalenko, Rainer F. Mahrt, Frank A. Nüesch, Jakob Heier

**Affiliations:** ^1^ Laboratory for Functional Polymers Empa Swiss Federal Laboratories for Materials Science and Technology Überlandstrasse 129 Dübendorf CH‐8600 Switzerland; ^2^ Institut des Matériaux École Polytechnique Fédérale de Lausanne EPFL Station 12 Lausanne CH‐1015 Switzerland; ^3^ Laboratory for Neutron Scattering and Imaging (LNS) Paul Scherrer Institute Villigen CH‐5232 Switzerland; ^4^ Laboratory of Inorganic Chemistry Department of Chemistry and Applied Biosciences ETH Zürich Vladimir Prelog‐Weg 1 Zürich CH‐8093 Switzerland; ^5^ Laboratory for Thin Films and Photovoltaics Empa Swiss Federal Laboratories of Materials Science and Technology Überlandstrasse 129, Dübendorf Zürich CH‐8600 Switzerland; ^6^ IBM Research–Zurich Säumerstrasse 4, Rüschlikon Zürich CH‐8803 Switzerland; ^7^ Present address: Department of Electrical and Systems Engineering University of Pennsylvania Säumerstrasse 4, Rüschlikon Philadelphia PA 19104 USA

**Keywords:** exciton lifetime, J‐aggregates, microemulsions, photoluminescence quantum yield, radiative excitons

## Abstract

Supramolecular assemblies from organic dyes forming J‐aggregates are known to exhibit narrowband photoluminescence with full‐width at half maximum of ≈9 nm (260 cm^−1^). Applications of these high color purity emitters, however, are hampered by the rather low photoluminescence quantum yields reported for cyanine J‐aggregates, even when formed in solution. Here, it is demonstrated that cyanine J‐aggregates can reach an order of magnitude higher photoluminescence quantum yield (increase from 5% to 60%) in blend solutions of water and alkylamines at room temperature. By means of time‐resolved photoluminescence studies, an increase in the exciton lifetime as a result of the suppression of non‐radiative processes is shown. Small‐angle neutron scattering studies suggest a necessary condition for the formation of such highly emissive J‐aggregates: the presence of a sharp water/amine interface for J‐aggregate assembly and the coexistence of nanoscale‐sized water and amine domains to restrict the J‐aggregate size and solubilize monomers, respectively.

## Graphical abstract

The narrowband photoluminescence quantum yield at room temperature of supramolecular assemblies of cyanine dyes (J‐aggregates) is enhanced from 5% to 60%. Confinement of J‐aggregates in a bicontinuous phase in water–alkylamine blends improves the quality of the J‐aggregates.

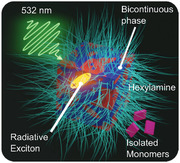

## 1 Introduction

Highly ordered supramolecular assemblies of organic dye molecules into 1D, 2D, and 3D structures (J‐aggregates) have a long history in science and technology^[^ ^1^ ^]^ and have recently attracted interest in the field of polariton lasers,^[^ ^2^ ^]^ organic light‐emitting devices,^[^ ^3^ ^]^ hybrid energy transfer systems,^[^ ^4^, ^5^, ^6^ ^]^ narrowband photodetectors^[^ ^7^, ^8^, ^9^ ^]^, and emerging opto‐excitonic switches.^[^ ^10^ ^]^ Strong coupling of transition dipole moments between dye molecules leads to specific optical properties such as narrow absorption and photoluminescence (PL) bands with small Stokes’ shift. It can be recognized though that many applications of J‐aggregate forming dyes are not making use of their PL properties, which may explain why only a limited number of research deals with that topic.^[^ ^11^ ^]^ This is despite the fact that luminescent J‐aggregates can open new perspectives in all fields where PL plays a role. The generally observed low PL quantum yield (PLQY) in J‐aggregates at room temperature was explained by static disorder, vibronic coupling, or non‐radiative relaxation of self‐trapped excitons.^[^ ^12^ ^]^ Among all organic J‐aggregate forming dyes, cyanine dyes stick out with characteristic features: high ground state polarizability, small bond‐length alternation, and charge inversion upon photoexcitation give the aggregates high structural stability and their unique spectral properties. Another valuable property of cyanine dyes is the possibility to extend the absorption range to the near‐infrared (NIR).

Numerous applications of cyanine J‐aggregates are currently investigated that would greatly benefit from an increased PLQY. An obvious example is a work on high color purity emitters.^[^ ^3^ ^]^ Water‐soluble J‐aggregates with PL in the short‐wavelength infrared (1000–2000 nm) region offer a suitable spectroscopic range for bioimaging.^[^ ^13^, ^14^ ^]^ Novel plasmonic effects are expected by plasmon–exciton coupling between metal or semiconducting nanostructures and J‐aggregates.^[^ ^15^ ^]^ So far research is only focusing on absorption and scattering effects.^[^ ^16^ ^]^ Long‐range exciton delocalization can be exploited in sensors (superquenching).^[^ ^17^ ^]^ Another application which requires high values of the PLQY is two‐photon PL microscopy for biological imaging.^[^ ^18^ ^]^ Furthermore, exploiting the high extinction coefficient of J‐aggregates as donor material in hybrid organic/inorganic systems (in particular, J‐aggregate/quantum dots, QDs) has shown to significantly improve the properties of the hybrid system,^[^ ^4^, ^5^, ^19^ ^]^ caused by the efficient Förster resonance energy transfer (FRET) process between the donor (J‐aggregates) and acceptor (QDs). The efficiency of non‐radiative FRET energy transfer depends inversely on the sum of radiative PL rate and non‐radiative decay rate of the donor in the absence of the acceptor. Hence, it is of paramount importance to minimize the non‐radiative recombination of excitons or, in other words, to increase the PLQY of the donor (i.e., J‐aggregates). For instance, Wang and Weiss^[^ ^20^ ^]^ have investigated the FRET rate between QD‐to‐J‐aggregates and J‐aggregates‐to‐QDs. The energy transfer yield for QD‐to‐J‐aggregates was ≈70%, while for J‐aggregates‐to‐QDs ≈25% was reported. Such low energy transfer efficiency from the J‐aggregates to QDs was ascribed to non‐radiative exciton decay in J‐aggregates competing with the FRET process. Decoupling exciton delocalization from the vibrationally excited ground state partially increases the PLQY due to reduced vibrational relaxation. Interestingly, the weak coupling of triplet excitons in J‐aggregates can also be used to decouple the electronically excited state from highly excited vibrational states of the ground state. This alleviates the commonly observed PL quenching which increases at longer emission wavelength, limiting the PL quantum efficiency of NIR emitters to 5–12%.^[^ ^21^ ^]^ Therefore, improving the PLQY by reducing the non‐radiative decay channels of J‐aggregates is necessary to enhance the performance of the hybrid systems discussed above.^[^ ^19^, ^20^, ^22^, ^23^, ^24^, ^25^ ^]^ Furthermore, emitters with ultra‐narrow PL spectra are of great importance in active pixel display technologies, where the narrow‐emission band could result in emitting devices with more brilliant colors. However, the low PLQY typical for regular J‐aggregates hampered their wide‐spread use in such devices, an opportunity that could come within reach once the PLQY is boosted to the level of common‐used organic emitting compounds, with PLQY above 50%.

J‐aggregates from a cyanine dye 5,6‐dichloro‐2‐[[5,6‐dichloro‐1‐ethyl‐3‐(4‐sulfobutyl)‐benzimidazol‐2‐ylidene]‐propenyl]‐1‐ethyl‐3‐(4‐sulfobutyl)‐benzimidazolium hydroxide, inner salt, sodium salt, famously known as TDBC have been used in hybrid light‐emitting devices^[^ ^3^, ^23^ ^]^ and hybrid energy transport systems.^[^ ^4^, ^24^ ^]^ The J‐aggregates formed from the TDBC dye exhibit a full‐width at half maximum (FWHM) in the absorption of 13 nm (393 cm^−1^) and PL of 9 nm (260 cm^−1^). For device applications, J‐aggregates have to be deposited as thin films, and the PLQY in thin films was reported to be ≈2%.^[^ ^23^ ^]^ Recently, we have shown that the absolute quantum yield can be improved by up to ≈5% at room temperature by growing high‐quality J‐aggregate crystals in thin films.^[^ ^26^ ^]^ However, the quantum yield still remained low, motivating us to revisit aggregate formation in solution. Grain boundaries,^[^ ^27^ ^]^ or stacking defects in the J‐aggregate crystal edges, self‐quenching^[^ ^28^ ^]^, and H‐aggregates^[^ ^29^ ^]^ that can quench excitons act as non‐radiative decay channels and are thought to limit the PLQY. Already small structural changes in the dye molecule led to different aggregate morphologies with different optical properties.^[^ ^30^ ^]^ It was shown that J‐aggregate PL is extremely sensitive to disorder and defect sites, and the reorganization of dye molecules on a nanometer scale can improve optical properties drastically.^[^ ^31^ ^]^ Strategies like the addition of surfactant indeed resulted in modification of morphology,^[^ ^32^ ^]^ micelle formation^[^ ^33^ ^]^, and a substantial increase in quantum yield of J‐aggregates (38% for monomethine dyes and 19% for trimethine dyes).^[^ ^34^ ^]^ Continued efforts to increase the PLQY of J‐aggregates by confining them into reverse micellar systems have shown only limited success.^[^ ^35^, ^36^, ^37^ ^]^ The above mentioned enhancement of the PLQY of cyanine dye J‐aggregates was caused by the formation of a “J‐aggregate‐surfactant” complex in solutions containing a surfactant above the critical micelle concentration.^[^ ^34^ ^]^ However, no clear correlation could be drawn between morphology modification and quantum yield improvement. To the present date, for comparison, J‐aggregates from perylene bisimide in solution is the only system with a near 100% PLQY at room temperature.^[^ ^38^, ^39^ ^]^ Here, we propose a new strategy for the formation of J‐aggregates from cyanine dyes with high PLQY (≈60% at room temperature). A blend solution of water and amines forms a nanoscale‐sized bicontinuous phase morphology. This morphology facilitates the stabilization of J‐aggregates at the amine/water interface while monomers are solubilized in the non‐polar environment. Time‐resolved PL (TRPL) studies reveal an increase in the lifetime of J‐aggregates together with an increase in PLQY.

To investigate the structural properties of the solutions that favored the formation of highly emissive J‐aggregates, we employed small‐angle neutron scattering (SANS). SANS is a very powerful tool to probe nanomorphologies in solution^[^ ^40^ ^]^ but can also reveal structural properties of J‐aggregates.^[^ ^32^ ^]^

## 2 Results

### 2.1 Optical Properties of J‐Aggregate in Solution

**Figure 1:**
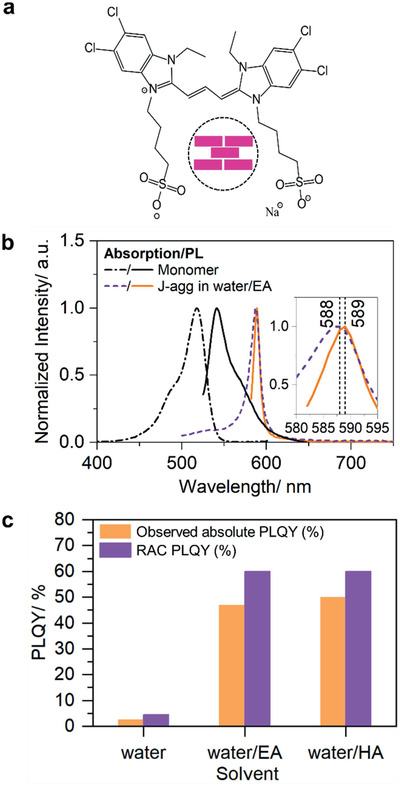
a) Chemical structure of the TDBC dye with a schematic showing the brickstone model of J‐aggregate packing from monomer molecules (in magenta). b) Absorption spectra for monomers in methanol and J‐aggregates in water/EA solution with their corresponding photoluminescence (PL) spectra recorded at excitation wavelength—520 and 532 nm, respectively. The inset is showing Stokes’ shift for J‐aggregates in water/EA. Compared to water, the spectra are slightly red‐shifted. c) Observed absolute PLQY and reabsorption corrected (RAC) PLQY for 1 mM J‐aggregate solutions in water, and blends of water/EA and water/HA with 50/50 ratios by volume. For statistics of the data, see Supporting Information.

The chemical structure of the TDBC cyanine dye used in this work is shown in **Figure** **1**a. Upon dissolving the dye in methanol, only monomers were present with peak absorption at 520 nm and emission at 541 nm. Water favors cyanine dye aggregation (brickstone model shown in Figure 1a), due to the high dielectric constant where repulsive forces between the charged molecules are reduced due to the charge screening effects. Indeed, dispersing the dye (*c* = 1 mM) in water led to the formation of J‐aggregates with peak absorption at 587 nm and emission at 588 nm (Figure 1b and Figure S1, Supporting Information). Adding 1 mL of ethylamine (EA) or hexylamine (HA) to 1 mL of the J‐aggregate solution immediately led to a significant PLQY increase as shown in Figure 1c. Moreover, adding EA to the aqueous J‐aggregate solution resulted in the emergence of the monomer phase without complete disintegration of the J‐aggregates (Figure S1, Supporting Information). To quantify these results, the PL of solutions of J‐aggregates in water, a 50/50 (vol%) blend of water/EA, and a 50/50 (vol%) blend of water/HA was recorded with an integrating sphere (see setup Figure S2, Supporting Information) at an excitation wavelength of *λ*_ex_ = 550 nm.

The observed absolute PLQY was first measured by following the method introduced by de‐Mello et al.^[^ ^41^ ^]^ and then the raw measurement value was corrected for reabsorption effects as reported by Ahn et al.^[^ ^42^ ^]^ to account for the small Stokes’ shift between J‐aggregate absorption and PL. The PLQY from J‐aggregates in water could be increased significantly from ≈4.5% to an absolute record value of ≈60.6% upon addition of amines (Figure 1c) while keeping the narrow FWHM (Figure S1b, Supporting Information). The PL peak slightly red‐shifts (by 91 cm^−1^) in the water/EA system compared to J‐aggregates in water. Furthermore, no additional PL peaks were observed at longer wavelengths as reported for other systems.^[^ ^43^ ^]^ The low‐temperature absolute PLQY was recorded by placing the sample (Figure S2, Supporting Information) in a glass dewar flask inside the integrating sphere and filled with liquid nitrogen. The absolute PLQY was continuously recorded as the sample cooled down from room temperature to 77 K. Upon cooling down to 77 K, the absolute PLQY values for J‐aggregates in water and water/EA was ≈3.7% (2.6% at 293 K) and ≈67.8% (46.9% at 293 K), respectively (Figure S3, Supporting Information). The increase in PLQY at low temperatures can be ascribed to the minimization of relaxation through non‐radiative decay channels and the emergence of superradiance (longer exciton coherence length) at cryogenic temperatures.^[^ ^44^, ^45^, ^46^, ^47^ ^]^ The PL excitation (PLE) spectra recorded for the J‐aggregates in water/EA solution fully overlaps with the absorption spectrum measured by UV–Vis spectroscopy, suggesting that the emission originates dominantly from the J‐aggregates (Figure S4, Supporting Information). Residual monomers within the Förster radius slightly contribute to the emission via an efficient energy transfer from monomer to J‐aggregates. Since the J‐aggregate/monomer ratio is very high in the PLE spectrum perfectly resembling the absorption spectrum, we can exclude direct emission from isolated monomers overlapping with the J‐aggregate emission.

**Figure 2:**
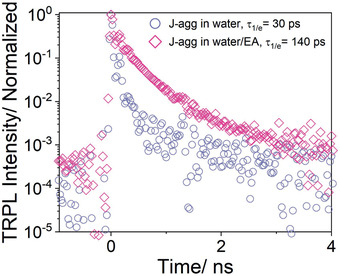
Spectrally integrated TRPL traces deconvoluted by the IRF. The TRPL raw data and IRF are shown in Figure S5, Supporting Information.

To get a deeper insight into the mechanism of the increase in the J‐aggregate's PLQY upon addition of EA, TRPL spectroscopy studies were performed. Here, we used J‐aggregate solutions in water and a water/EA system in cuvettes with 15 µm optical path length to avoid artifacts of reabsorption. A picosecond laser running at 532 nm wavelength with 20 MHz repetition rate was used for excitation. The TRPL data acquired by the detector after filtering the light through a monochromator are shown in Figure S5, Supporting Information. TRPL decay traces shown in **Figure** **2** for both systems exhibit a non‐exponential decay profile at room temperature. The experimental time‐resolved traces have been deconvoluted with the instrumental response function (IRF), shown in the Supporting Information, using the Richardson–Lucy algorithm to be model‐independent.

The exciton lifetime, defined here as 1/e decay time, is about 140 ps for J‐aggregates in water/EA compared to an exciton lifetime of 30 ps for J‐aggregates in water. The concomitant increase in a lifetime with the PLQY for J‐aggregates in the water/EA system is still below the monomer lifetime (2.4 ns) as calculated for the same dye from the Strickler–Berg relation.^[^ ^48^ ^]^ Here, a suppression of non‐radiative processes results in increased exciton lifetime and increased PLQY, as observed in our experiments.

Muneter et al.^[^ ^49^ ^]^ have investigated the influence of the J‐aggregate size on PL lifetime and quantum yield. Using J‐aggregates as donor and silver bromide (AgBr) as acceptor, the electron transfer process upon excitation of J‐aggregates was investigated. They reported that with an increase in aggregate size, funneling of excitons to non‐radiative decay channels becomes a dominant process. Also, small J‐aggregates are expected to have a higher efficiency of electron transfer from J‐aggregates to the AgBr conduction band. Taking this size effect of J‐aggregates onto the PLQY into account, it seems that the addition of EA to J‐aggregate solution reduces the physical size of J‐aggregates, thereby minimizing the non‐radiative decay channels (grain boundaries, defects) present in large J‐aggregate crystals (for example, J‐aggregates in water).

The coherence length *N*_c_ of the aggregate is the ratio of monomer radiative lifetime to aggregate radiative lifetime *N*_c_ = *τ*_rad,mon_/*τ*_rad,agg_^[^ ^1^ ^]^ and can be approximated from the data. With *τ*_rad,agg_ = *τ*_1/e_/PLQY = 0.23 ns and with the monomer lifetime estimated above we obtain *N*_c_ = 2.4 ns/0.23 ns = 10.43 at room temperature. The physical size of the aggregate must thus be at least 10 monomers.

Exciton–exciton annihilation is a well‐known process in J‐aggregates that shortens the lifetime. Given the low excitation‐fluence, we can safely neglect this process in our system (see Supporting Information).

Highly emissive J‐aggregates were observed in blends of water with EA, however only within certain ranges of ratios of water to amine. EA is supplied as an aqueous solution (70 wt% in water) in which the monomer is soluble. Addition of 1 mL EA to 1 mL aqueous J‐aggregate solution (50/50 vol% of water/EA) turns the “regular” J‐aggregate into a highly emissive J‐aggregate. Above 50 vol% of EA, only monomers were present. Throughout the entire probed phase space, the solution was homogeneous. In the water/HA system, the situation is quite different: only in blends where the HA content is below 5 vol% or is above 50 vol% (below 1 vol% or is above 50 vol% for a 1 mM dye content), the system remained in a single phase. Between 5 and 50 vol% the system phase‐separated into two macroscopic phases. In the single‐phase region (HA rich side) dye existed only as monomer, in the two‐phase region, emissive J‐aggregates formed and segregated to the top phase, while the bottom phase remained clear (Figure S6, Supporting Information).

The formation of highly emissive J‐aggregates did not depend on the sequence of the sample preparation. Adding dye to blend solutions of water and amines resulted in a similar PLQY.

### 2.2 Structural Properties of the D_2_O/HA Blend

Qualitatively, the phase behavior of a water/HA blend behaved similarly to the J‐aggregate/water/HA blend with an extended two‐phase region at the water‐rich side. To gain a better understanding of the role of amines in the stabilization of highly emissive J‐aggregates, we have probed the phases of the water/HA blend with SANS. From here on, all experiments were performed with deuterated water (D_2_O) to minimize background scattering in the neutron scattering experiments. Earlier work reported the phase diagram of the water/HA blend and pointed to the structure formation properties of amines in aqueous solutions, but to the best of our knowledge, no studies on the nano‐morphology are available.^[^ ^50^ ^]^

**Figure 3:**
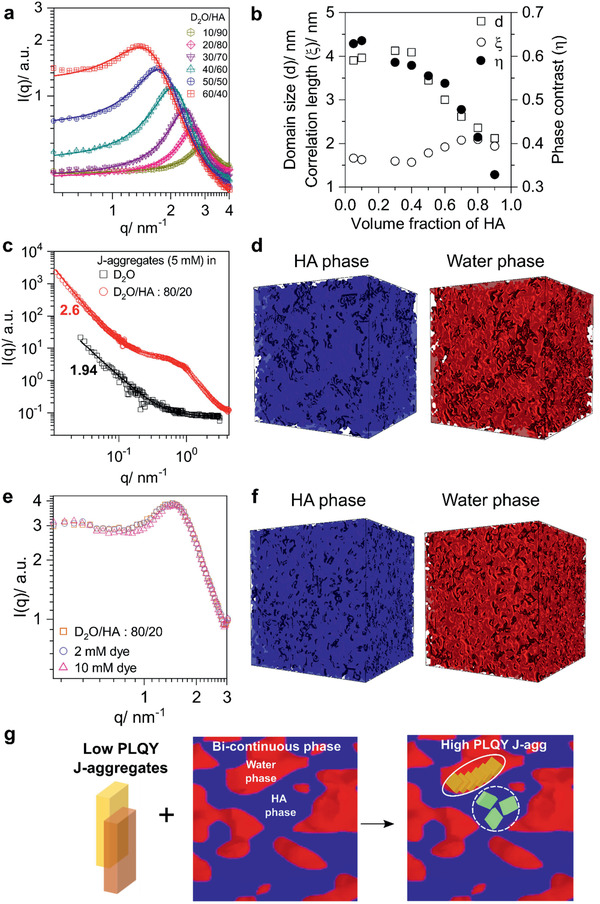
SANS spectra of D_2_O/HA blend samples (symbols) and fits with the Teubner–Strey model (solid lines) are shown in (a). The fitting parameters: scattering length density difference (*η*), characteristic domain size (*d*), and correlation length (*ξ*) are plotted in (b). SANS spectra and fitting functions of 5 mM J‐aggregate solutions in water and after adding c) HA and 3D representative morphologies of the HA phase (blue) and water phase (red) generated using d) SAXSMorph. The two phases complement each other. e) SANS spectra from the top phase of an 80/20 D_2_O/HA blend and the extracted top phase from the same blend after having added dye to it. f) 3D representative morphologies of solutions with a 10 mM dye content. The edge length of the cubes is 25 nm. g) Schematic showing high PLQY J‐aggregates formation at the water/HA interface (solid circle) devoid of defect molecules dissolved in HA (dotted circle).

In the single‐phase region (HA‐rich side), all spectra could be reproduced with high fidelity with the Teubner–Strey model^[^ ^40^ ^]^ for microemulsions (**Figure** **3**a,b and Section S1, Supporting Information). The Teubner–Strey model reproduces a single broad peak representing the alternating arrangement of two phases without long‐range order and a *q* ^−4^ (*q* is the magnitude of the scattering vector) dependence at large *q* attributed to a well‐defined internal interface, using only the three fitting parameters *d* (characteristic domain size), *ξ* (correlation length), and *η* (scattering length density difference). In the single‐phase region, the domain size decreased from 3.45 to 2.11 nm with increasing HA content (Table S1, Supporting Information). In the phase‐separated systems (i.e., D_2_O/HA: 80/20), the top phase was carefully extracted to perform SANS measurements. All SANS spectra of the top phase (D_2_O/HA: 95/05 to 60/40) looked similar and could be fitted reasonably well with the Teubner–Strey model, with domain sizes around 4 nm (Figure 3b). Alternatively, we also analyzed the data with SAXSMorph, a program that generates a model‐independent “representative” morphology from the data.^[^ ^51^ ^]^ A bicontinuous structure with domain connectivity above 97% was found (Figure S7, Supporting Information). For the bottom phase, SANS did not reveal any characteristic features.

Also in other respects, the blend behaved like a microemulsion: overtime no coarsening was observed. The observed composition dependence of the water droplet size is typical for an inverse microemulsion system.^[^ ^52^ ^]^ In a narrow composition range of D_2_O/HA around 90/10, a separation into three macroscopic phases was observed, typical for microemulsions, whereby the middle phase is assigned to a bicontinuous phase.^[^ ^53^ ^]^

It appears surprising that we can form a microemulsion from a binary blend without surfactant and co‐surfactant. However, the “quality” of a microemulsion can be assessed with the help of the amphiphilicity factor, *f*_a_ (Table S1, Supporting Information).^[^ ^54^ ^]^ A strongly structured, “good” microemulsion has an amphiphilicity between −1 < *f*_a_ < 0; while for “poor” microemulsions values between 0 < *f*_a_ < 1 are found.

Therefore, the microemulsions formed by the D_2_O/HA system are of relatively poor quality, with the top phase in the two‐phase region having slightly higher amphiphilicity. To this end, we have extensively investigated the morphology of the D_2_O/HA system for a wide composition range. In the next section, we describe the ternary system D_2_O/HA/dye for one composition (D_2_O/HA: 80/20) which exhibits phase separation.

### 2.3 Structural Properties of J‐aggregates in the Solution

SANS spectra and fits to the experimental data for different J‐aggregate solutions are presented in Figure 3c,e and Figure S8 and S9, Supporting Information. We investigated solutions of J‐aggregates in water, J‐aggregates in water after adding HA following the experiments of Section 2.1, and solutions made with a different sequence: dye was added to a D_2_O/HA blend after the microemulsion had formed. The SANS spectra of J‐aggregates in D_2_O were fitted with a model of a Porod‐like background by the function *I(q) α q^−*α*^* with an exponent *α* = 2.16 and 1.94 (0.2 and 5 mM J‐aggregate solution, respectively). Such scaling behavior is characteristic of objects that can be described as mass fractals, where mass fractal scaling can be associated with the packing efficiency of particles.^[^ ^55^ ^]^ A value of *α* = 2 is typical for a thin (2D) plate. For more complex morphologies such as branched objects, also non‐integer numbers are observed. The curves showed mass scaling with constant slope down to the lowest detectable *q*‐range, indicating that the aggregates are larger than the 300 nm probed by SANS.^[^ ^56^, ^57^ ^]^ This observation is consistent with scanning probe microscopy investigations of J‐aggregates adsorbed from solution onto a substrate, which confirmed crystalline domains larger than 500 nm.^[^ ^26^ ^]^ When dye was added to the extracted top phase of a water/HA blend (starting vol% 80/20), emissive J‐aggregates formed, but the SANS spectrum did almost not change over the water/HA blend (Figure 3e). More remarkably, no signature of a mass fractal object of a large dimension could be identified in the spectrum. The representative morphology suggested by SAXSMorph showed a bicontinuous structure (Figure 3f). The morphology did not differ notably from the morphology that had formed before adding dye (Figure S7, Supporting Information).

Differently, upon adding HA to an aqueous solution in which poorly emissive J‐aggregates had already formed (up to volume fractions of D_2_O/HA = 80/20), phase separation occurred (Figure S6, Supporting Information), the J‐aggregate depleted the bottom phase and transformed into its emissive state in the top phase, the SANS spectrum of the top phase showed now very different features (Figure 3c). Enhanced scattering in the mid *q*‐range was observed, indicating local ordering. In the high *q*‐range, no features characteristic to microemulsion formation were visible, while in the low *q*‐range a power‐law is dominant.

Good fits to the scattering intensity profile were possible with different models that were rather insensitive to the exact form factors. For that reason, we here only applied SAXSMorph for data analysis. The routine was applied to the original data after subtraction of background and power‐law with scaling exponent of *α* = 2.6 (Figure S9, Supporting Information). From the visual qualification, we find a bicontinuous structure with few isolated domains (Figure 3d). At first sight, the structure resembles the features of the sample D_2_O/HA: 80/20 blend after adding dye (Figure 3f). We expected at least some similarity, as in both samples the dye assembled into the emissive J‐aggregate. But the morphologies also must differ enough to account for the different scattering functions. This difference becomes most easily visible when looking at a cross‐section of the 3D morphologies (Figure S10, Supporting Information). The features of the D_2_O/HA: 80/20 10 mM dye sample are uniform and dominated by one length scale *λ* (the peak at *q* = 1.48 nm^−1^, corresponding to *λ* = 4.26 nm, Figure 3e). In the J‐aggregate D_2_O/HA: 80/20 sample a superordinated structure separates a phase with a higher volume fraction of the HA phase from a phase with a lower volume fraction of the HA phase. The existence of two length scales is also anticipated from the scattering function, which shows a hump at *q* = 0.53 nm^−1^ and *q* = 0.87 nm^−1^, corresponding to *λ* = 12.0 nm and *λ* = 7. 26 nm, respectively, Figure 3c). We believe that the 12.0 nm feature is related to the interface assembly of J‐aggregates, we estimated the J‐aggregate size to be above 10 nm.

## 3 Discussion

We here discuss in more detail the role of the matrix morphology and the role of the amine functional endgroup. To link the formation of emissive J‐aggregates to the microemulsion morphology, it is most instructive to look at the water/HA blend as this blend shows a large variety of morphologies which we probed with SANS (Figure 3b). We proved that emissive J‐aggregates only formed in a bicontinuous but not in a droplet phase. In the two‐phase region dye or J‐aggregates leave the water‐rich region and segregated to the bicontinuous microemulsion phase, where they formed emissive aggregates (Figure S6, Supporting Information). Adding dye to a simple immiscible interfacial system as described for example by the *Gibbs Dividing Surface*,^[^ ^58^ ^]^ would lead to a homogeneous dye distribution in the HA phase and an enriched dye region at the interface, as the dye is poorly soluble in water while it is soluble in amines. With the surface tensions of water and HA of 72 mN m^−1^ and 27.3 mN m^−1^, respectively, and the slightly amphiphilic character of the dye TDBC with hydrophilic (—C_3_H_6_SO_3_) side chains, and hydrophobic conjugated pi system and (—C_2_H_5_) side chains, adsorption at the water/HA interface can be expected (where it will aggregate) as the dye can lower the overall interfacial energy.

The morphology in a microemulsion is dominated by a large number of interfaces. Furthermore, it is reasonable to conclude that bicontinuous phases with regions of null curvature at the interfaces allow for aggregation, while the droplets are too small to incorporate an aggregate, and the interfaces show a too high curvature to assemble dye in a J‐aggregate fashion. Furthermore, the phase‐contrast between the domains was larger, and the amphiphilicity factor was smaller, indicating a “better” microemulsion with better‐defined interfaces.

Earlier strategies to increase the PLQY of cyanine dye J‐aggregates included defect‐free assembly on micelles or size‐confinement in water droplets^[^ ^34^ ^]^ but none of these strategies had been crowned with success. For that reason, we believe that the good solubility of the non‐aggregated dye species in one of the phases of the microemulsion is an additional necessary condition to boost the PLQY to the high values observed here. Dye monomers are soluble in HA and are in thermodynamic equilibrium with dye assembled at the interface. Loosely bound dye molecules that introduce defects and cause exciton quenching sites are thus easily dissolved in the alkyl environment in the neighboring HA phase (Figure 3g).

To summarize our model: necessary conditions for the formation of emissive J‐aggregates is the concurrence of several properties found in the microemulsion: defined interfaces along which the dye assembles, areas of low curvature as can be found in bicontinuous structures, and good solubility of dye monomers in one of the components of the microemulsion.

The SANS experiments support the model described above. Adding non‐aggregated dye molecules to the microemulsion as described first, did not alter the SANS spectrum (Figure 3e), the overall morphology was thus mainly controlled by the microemulsion. This is not too surprising given that even at a 5 mM dye concentration, the number of dye molecules in the blend is low (3 × 10^—3^ molecules nm^—3^) compared to the density of domains in the microemulsion. From here it can also directly be concluded that the emissive J‐aggregates were small in size, they did not significantly contribute to the neutron scattering intensity and no size scaling at low *q*‐values was observed.

In the second experiment, amines were added to a J‐aggregate solution. This also led to emissive J‐aggregates, but with a different scattering function, which indicated a different morphology. The addition of amines did not disintegrate the large J‐aggregates but alter their fractal behavior from a pure 2D structure (*α* = 1.94) to a network structure (*α* = 2.6), and the overall scattering intensity at low *q* increased significantly (Figure 3c). The increase in scattering intensity was caused either by an increase in scattering volume or by an increase in scattering contrast between J‐aggregate and D_2_O. The origin of the latter is an attachment of HA to the J‐aggregate, driven by electrostatic interactions between J‐aggregates and amines (see values for scattering length density in Table S3, Supporting Information).^[^ ^60^ ^]^

The process for structure formation could be as follows: upon addition of HA, water/HA interfaces were forming and sweep through the solution that contained large 2D J‐aggregates. Branching points, grain boundaries, and loosely bound molecules were prone to HA attack because of the good solubility of the dye in HA. “Purified” J‐aggregates then assembled at the newly formed interfaces, either from fragments of larger J‐aggregates or from dye monomers.

No conclusions could be drawn on the exact arrangement of dye molecules as the resolution of SANS is not sufficient to resolve molecular packing, but it can be safely concluded that the emissive J‐aggregates are significantly smaller in size than the TDBC J‐aggregates forming in water.

Even though SANS does not “image” a morphology, a bicontinuous microemulsion (as opposed to a droplet morphology) was identified as base morphology for emissive J‐aggregate formation. We anticipate that the remaining large J‐aggregates are non‐emissive.

We finally look at the role of the amine endgroup. In a previous study,^[^ ^26^ ^]^ we had grown J‐aggregates of the same TDBC dye on amine‐functionalized glass substrates, demonstrating the stability of the J‐aggregate/amine interface. Similarly, Bradley et al.,^[^ ^59^ ^]^ have demonstrated the formation of J‐aggregates on amine‐functionalized (PDAC) cationic polyelectrolytes. More evidence for the formation of a J‐aggregate–amine complex is given in Section S2, Supporting Information, where we describe the behavior of J‐aggregates upon addition of 5 vol% HA. A large structural change was observed in the mid‐Q‐range. Even though the scattering function did not show the typical features of a microemulsion, also here a bicontinuous morphology developed as shown in the reconstruction with SAXSMorph (Figure 3c).

In search for a general law for the formation of emissive J‐aggregates, we screened a number of different amines (Table S4, Figures S11 and S12, Supporting Information). The trend is very clear: only the alkylamines support the formation of emissive J‐aggregates. A detailed morphological study though was only performed with the water/HA system.

Similarly, we screened a number of cyanine dyes to evaluate their aggregation behavior in a water/HA emulsion. None of the tested dyes formed emissive J‐aggregates (Figure S13, Supporting Information). This observation is not surprising as J‐aggregation is a very delicate process in which small modifications largely affect the final supramolecular assembly.

## 4 Conclusion

The uniqueness of J‐aggregates can be envisaged from the number of expressions that were created to describe their properties (“superradiance,” “superquenching,” “supersensitizing,” “superblinking”). Still, a lot of work is ahead of us to exploit their potential PL properties in technical applications. The challenge is not only in understanding the correlation between molecular structure of the cyanine and the conditions that lead to aggregation but also to control aggregate size and susceptibility to the formation of defects. The present work is a first step in this direction: the dye TDBC is a frequently studied dye in many contexts. Spontaneous self‐assembly of cyanine dye molecules in water into large J‐aggregate microcrystals introduces crystallographic defects which non‐radiatively quenched excitons, leading to a low PLQY. By blending the dye with a microemulsion where the interfaces are dictating aggregate formation and size, the PLQY could be increased by one order of magnitude. Morphological disorder leading to non‐radiative decay pathways can be greatly reduced when aggregation occurs in a more controlled manner dictated by the microemulsion. Next to size control, grain boundary passivation must play a role that does not allow monomers to add and form exciton quenching sites. The appeal of our system is how straightforward the experiment can be performed. For future work, we believe that understanding the dissolution of dye, dipole–dipole interaction and packing of dye molecules in amine/water interface will benefit in achieving high PLQY in other dyes, considering the large number of microemulsions that are known.

## 5 Experimental Section

##### J‐aggregate Preparation

The cyanine dye (5,6‐dichloro‐2‐[[5,6‐dichloro‐1‐ethyl‐3‐(4‐sulfobutyl)‐benzimidazol‐2‐ylidene]‐propenyl]‐1‐ethyl‐3‐(4‐sulfobutyl)‐benzimidazolium hydroxide, inner salt, sodium salt (TDBC)) procured from FEW Chemicals, Germany was used without further purification. EA solution (70 wt% in H_2_O) and HA were purchased from Sigma Aldrich. The TDBC dye was dissolved in Millipore water to form J‐aggregates. After an idle period of 24 h, the J‐aggregate solution was mixed with EA and HA in different ratios. For SANS experiments, deuterium oxide (D_2_O, 99.9%, Cambridge Isotope Laboratories, Inc.) was used instead of water.

##### Optical Properties

Absorption of the J‐aggregate solution was measured using a Varian Cary 50 UV–Vis spectrophotometer. PL of the dye solution was measured using glass capillaries with 1 mm path length to minimize reabsorption in an integrating sphere. The absolute PLQY was measured using a Quantarus‐QY C11347 spectrometer from Hamamatsu followed by reabsorption correction as reported by Ahn et al.^[^ ^42^ ^]^ In order to minimize self‐absorption, we used, when possible, less optically dense samples, special homemade thin (15–50 µm) cuvettes, thin (1 mm) capillaries, or applied reabsorption correction methods approved by equipment producer (Hamamatsu for PLQY measurement). Briefly, the dye solution taken in the glass capillary was placed in the integrating sphere to record the emission spectrum (F_obs_(*λ*), including reabsorption). The emission spectrum for the same solution was recorded without integrating sphere (F′(*λ*)). The reabsorption factor (a) was calculated using, $\int_{0}^{\infty }F_{\mathrm{obs}}\left(\lambda \right)d\lambda /\int_{0}^{\infty }F^{\prime }\left(\lambda \right)d\lambda =\;1-a.\;$The reabsorption factor is used to correct the observed quantum yield (QY_obs_) as QY  = QY_obs_ /(1 − a). In addition, we used rhodamine 101 as a standard to confirm the accuracy of our measurement.^[^ ^61^ ^]^ Our PLQY value (86%) slightly deviates (<10%) from the reported value (91%). PLE spectra for the J‐aggregate emission wavelength were acquired in the Fluorolog spectrometer with an emission wavelength setting at 600 nm and monochromator slit settings of 0.80 nm at light source and detector.

##### Time‐Resolved Photoluminescence (TRPL)

A frequency‐doubled picosecond Nd:YAG laser operating a 532 nm with 20 MHz repetition rate was used to excite the sample (average power of 2.2 W cm^−2^). The room temperature TRPL for the J‐aggregates in water and water/EA solution was studied by time‐correlated single‐photon counting using PicoQuant FluoTime 300 fluorescence lifetime spectrometer (time resolution ≈ 150 ps). To minimize the reabsorption of emitted photons we used dye solutions (*c* = 1 mM) in cuvettes with path length ≈ 15 µm for TRPL measurements.

##### Small‐Angle Neutron Scattering (SANS)

The SANS experiments were carried out at the SINQ neutron source at PSI, Villigen, Switzerland. The measurements were carried out with a neutron wavelength of 1.6 nm, a 2D position‐sensitive detector of size (96 cm × 96 cm) at sample to detector distances of 1.6 to 18 m where the scattering wave vector (*q*) could be scanned in the range of *q* = 0.01*–*4.27 nm^−1^.^[^ ^60^ ^]^ Solutions were filled in quartz cuvettes with a path length of 2 mm. A cuvette filled with deuterium oxide (D_2_O) was used for baseline correction. Morphologies were generated with SAXSMorph,^[^ ^51^ ^]^ and plotted with the POV‐Ray software.

##### Statistical Analysis

To validate the significant difference in PLQY values for the water and water/EA systems, we performed six independent measurements. Standard deviations were 0.157 and 3.022, respectively. Using the Graphpad Prism 8.4.3. software, the observed absolute PLQY values were then analyzed using the two‐tailed unpaired *t*‐test. The very small p‐value of *p* < 0.0001 confirms that the difference between the measured PLQY in water and water/EA was unlikely to be caused by measurement error or chance. According to the manufacturer, the set‐up accuracy for the PLQY measurement was supposed to be better than 3% and the measurement accuracy better than 1%.

All SANS spectra were measured with a minimum total number of counts of 10^6^ for sufficient statistics. Data reduction was performed with the program BERSANS,^[^ ^62^ ^]^ which generates and calculates values of the statistical error from the 2D dimensional “raw data.” Details on error calculation were described in the BERSANS manual. In Figure S14, Supporting Information, we show representative data including error bars. The errors were only significant for the low and high *q*‐values for each detector setting.

Fits to the SANS data were performed with SASfit,^[^ ^63^ ^]^ the *χ* ^2^‐values were minimized, goodness‐of‐fit parameters were calculated following that. We use the *R*‐factor introduced by crystallographers^[^ ^64^ ^]^ as a measure for the goodness of a fit. The *R*‐factor was defined as

(1) $$R\;=\;\frac{\sum_{t\;=\;1}^{N}\left|\left|I_{\exp}\left(q_{t}\right)\right|-\left|I_{\mathrm{th}}\left(q_{t}\right)\right|\right|}{\sum_{t\;=\;1}^{N}\left|I_{\exp}\left(q_{t}\right)\right|}$$

whereby *I*_exp_ and *I*_th_ were the experimental and theoretical scattering intensities. Possible values range from zero for perfect agreement of model and observation to infinity, whereby *R* values < 0.05 were considered to be good fits. Table S1, Supporting Information, displays the *R*‐values for data fits to the microemulsions, following the criteria from above the fits were very good.

The program SAXSmorph which we used to generate a model‐independent morphology was a software, which applies the clipped random wave theory to small‐angle scattering data to obtain the two‐point correlation function, and spectral function, which was done by a numerical inversion of the integral equations. The numerical inversion does not supply an error bar of these functions, but the quality of the inversion can be tested by the theoretical scattering curve based on the calculated power spectrum or two‐point correlation function. The comparison of the theoretical and experimental curves then could be done via the *R*‐factor.

## Conflict of Interest

The authors declare no conflict of interest.

## Supporting information

Supporting Information
