# Supplementary material for: Enhanced Room‐Temperature Photoluminescence Quantum Yield in Morphology Controlled J‐Aggregates
Source: Adv Sci (Weinh). 2021 Jan 4;8(4):1903080. doi: 10.1002/advs.201903080 (PMC7887577; doi:10.1002/advs.201903080)
Supplement: Supplementary file 1 — Supporting Information [file ADVS-8-1903080-s001.pdf]

WILEY-VCH

© 2020 Wiley-VCH GmbH

ADVANCED SCIENCE
Open Access

Supporting Information

for *Adv. Sci.,* DOI: 10.1002/advs.202003080

Enhanced Room Temperature Photoluminescence Quantum Yield in Morphology-controlled J-aggregates

Surendra B. Anantharaman,[†] Joachim Kohlbrecher, Gabriele Rainò, Sergii Yakunin, Thilo Stöferle, Jay Patel, Maksym Kovalenko, Rainer F. Mahrt, Frank A. Nüesch, and Jakob Heier**

© 2020 Wiley-VCH GmbH

Supporting Information

Enhanced Room Temperature Photoluminescence Quantum Yield in Morphology-controlled J-aggregates

Surendra B. Anantharaman,,† Joachim Kohlbrecher, Gabriele Rainò, Sergii Yakunin, Thilo Stöferle, Jay Patel, Maksym Kovalenko, Rainer F. Mahrt, Frank A. Nüesch, and Jakob Heier**

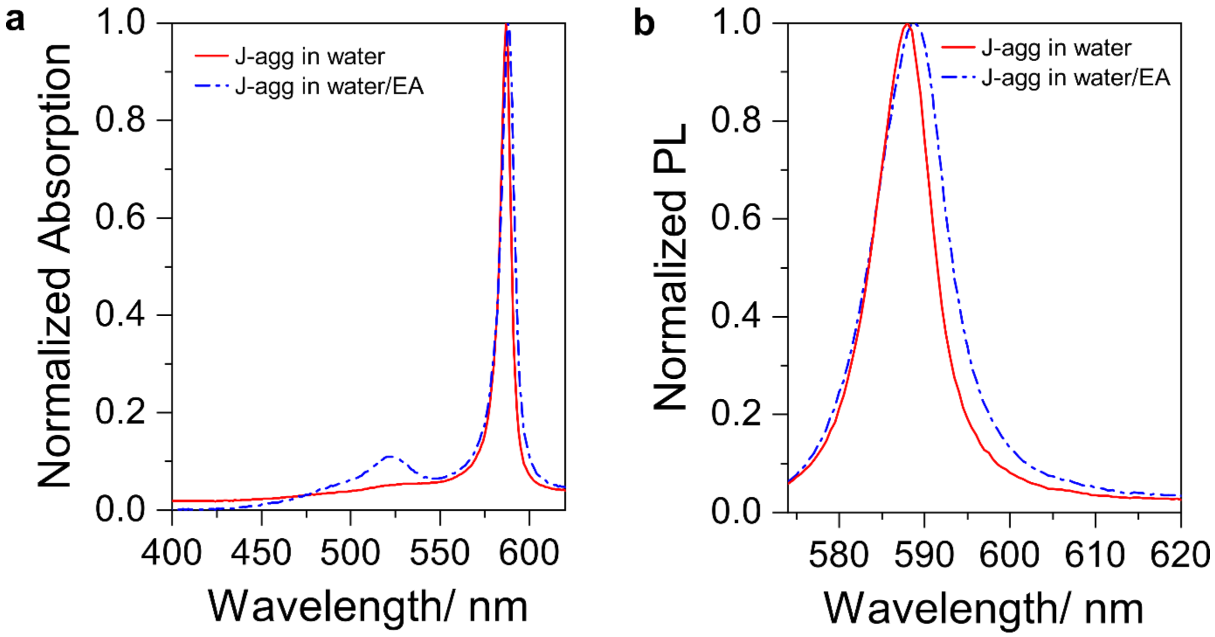

Figure S1. Normalized absorption (a) and PL recorded with an excitation wavelength of 532 nm (b) for J-aggregates in water and water/EA recorded using 15 μm path length cuvettes.

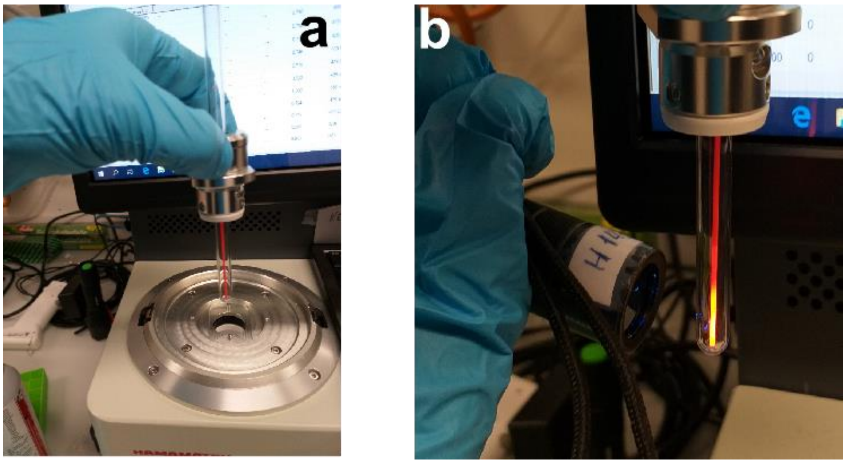

Figure S2. Absolute photoluminescence quantum yield measurement set-up using an integrating sphere with J-aggregate in water/EA solution taken in 1mm glass capillaries (a) and exposed to UV light (b).

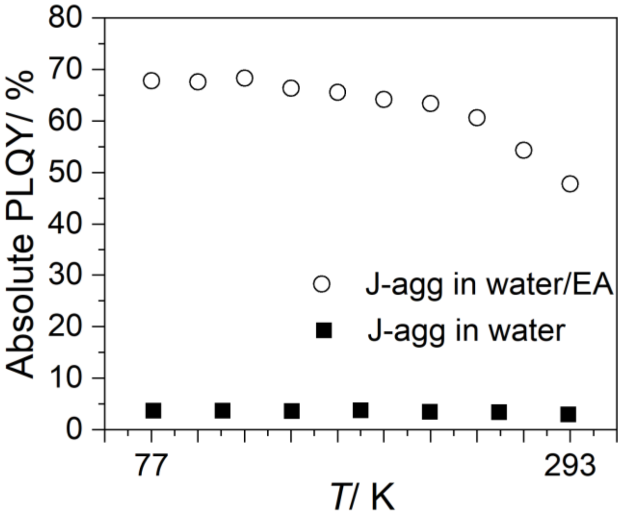

Figure S3. The absolute PLQY for J-aggregates in water and water/EA was continuously recorded while cooling the sample down from 293 K to 77 K.

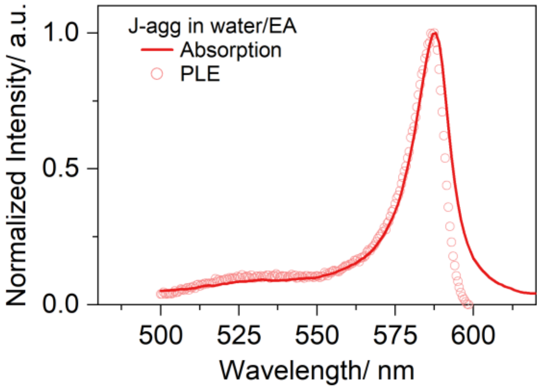

Figure S4. The absorption spectrum and photoluminescence excitation (PLE) spectrum for the PL emission wavelength = 600 nm measured from J-aggregates in water/EA.

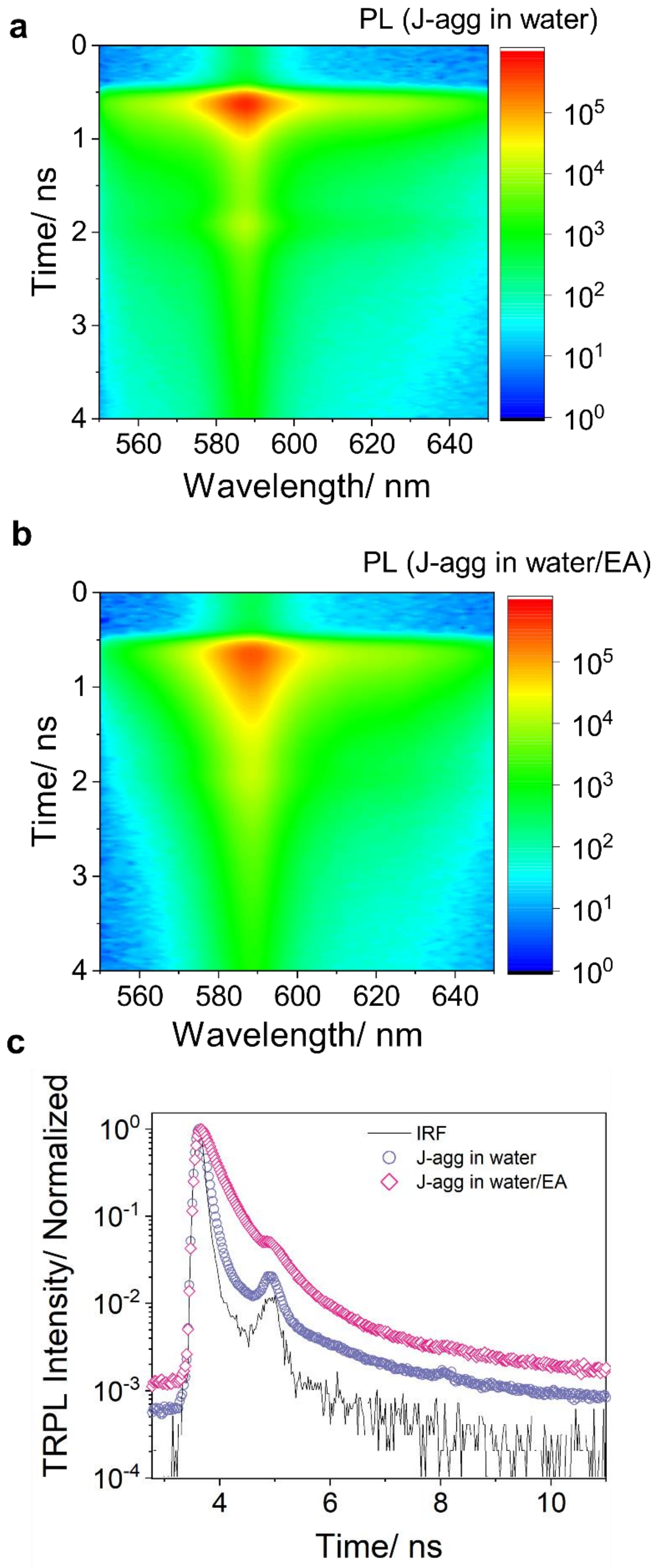

Figure S5. Energy- and time-resolved traces in a 2D-colored plot for J-aggregates in water (a) and water/EA (b) solution. TRPL raw data for J-aggregates in water and water/EA system along with the instrument response function (IRF) are shown in (c). The deconvoluted traces are shown in the main text (Figure 2).

Exciton-exciton annihilation

Exciton-exciton annihilation is a non-radiative interaction between two excitations, leading to an additional de-population of the excited state and thus a change in fluorescence lifetime.[1] We here present a quick estimate if exciton-exciton annihilation needs to be considered in our system. The laser is operated at 532 nm with 20 MHz repetition rate with an average power of 2.2 W / cm^2. Each laser pulse would carry an energy of E = 110 nJ over an area of 1 cm^2, which corresponds to $n = 3 \times 10^{11}$ photons / cm^2. At a lifetime of 140 ps and a laser repetition rate of 20 MHz, we can safely assume that we only need to look at one pulse. The samples were probed in a 1.5 μm thick cuvette, which would average to $N = 9 \times 10^{14}$ molecules / cuvette on an area of 1 cm^2 (1 mM solution). With an absorption cross section of a dye molecule $\sigma = 6.25 \times 10^{-17}$ cm^2,[1] and an estimated coherence length of N = 10 molecules, the laser power is by far not sufficient to induce exciton-exciton annihilation.

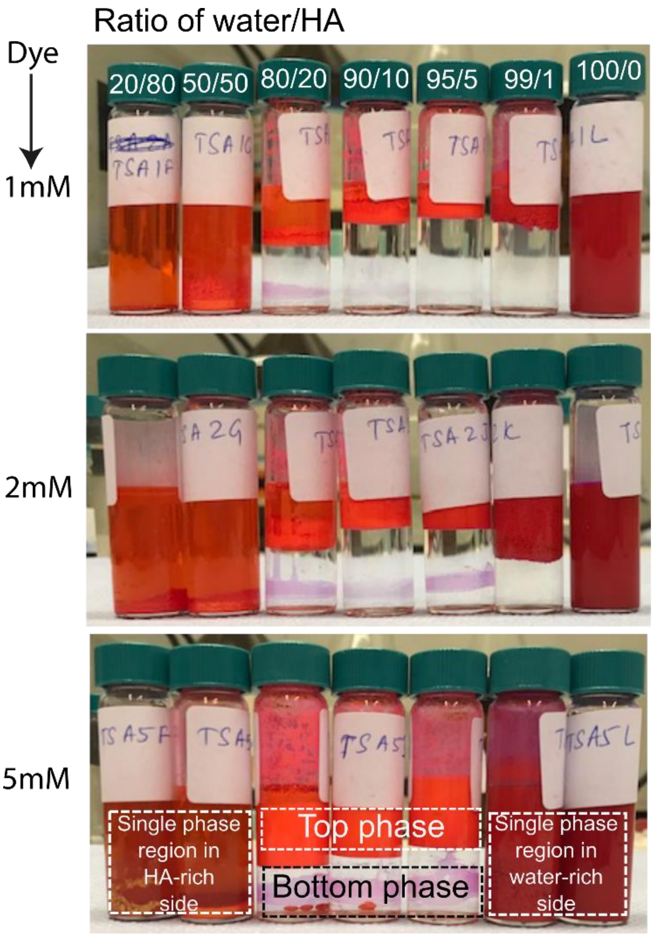

Figure S6. J-aggregate solutions in water/HA for different dye concentrations. The solubility of dye in water/HA : 20/80 and 50/50 is very low, precipitates of excess dye are also visible in the monomer phase.

Section S1: Small Angle Scattering on Microemulsions, Teubner-Strey formula

Teubner and Strey obtained the static scattering intensity distribution I(q) of micro-emulsions from a Landau theory.[2] The formula takes the following form:

$$I(q) = \frac{8\pi\langle\eta^2\rangle/\xi}{a^2 - 2bq^2 + q^4} \qquad \text{(S1)}$$

with $\langle\eta^2\rangle$ being the mean square fluctuation of the scattering density and q is the scattering vector. The correlation length of the fluctuations ξ is derived from the correlation function

$$\gamma(r) = \frac{d}{2\pi r} e^{-\frac{r}{\xi}} sin\left(\frac{2\pi r}{d}\right) \qquad \text{(S2)}$$

and $a = k^2 + 1/\xi^2$, $b = k^2 - 1/\xi^2$.

Table S1: Fitting parameters characteristic correlation length (ξ), the domain size (*d*), and the scattering length density difference (η) and R-factor (goodness of fit) to the Teubner-Strey model for different D_2O/HA blend samples. The amphiphilicity factor (f_a) is calculated from the fitting parameters. In phase separated samples (D_2O/HA = 90/10, 70/30, 60/40) only the top phase was measured. The spectra and fitting parameters should be similar as the separated top phase has a composition around D_2O/HA = 50/50.

D_2O/HA	ξ / nm	*d* / nm	η	f_a	R
90/10	1.628	3.964	0.636	0.74	-
70/30	1.589	4.123	0.587	0.71	-
60/40	1.568	4.090	0.579	0.71	-
50/50	1.783	3.450	0.555	0.83	0.030
40/60	1.918	2.996	0.538	0.88	0.018
30/70	2.076	2.611	0.478	0.92	0.015
20/80	2.086	2.352	0.414	0.94	0.013
10/90	1.939	2.114	0.328	0.94	0.010

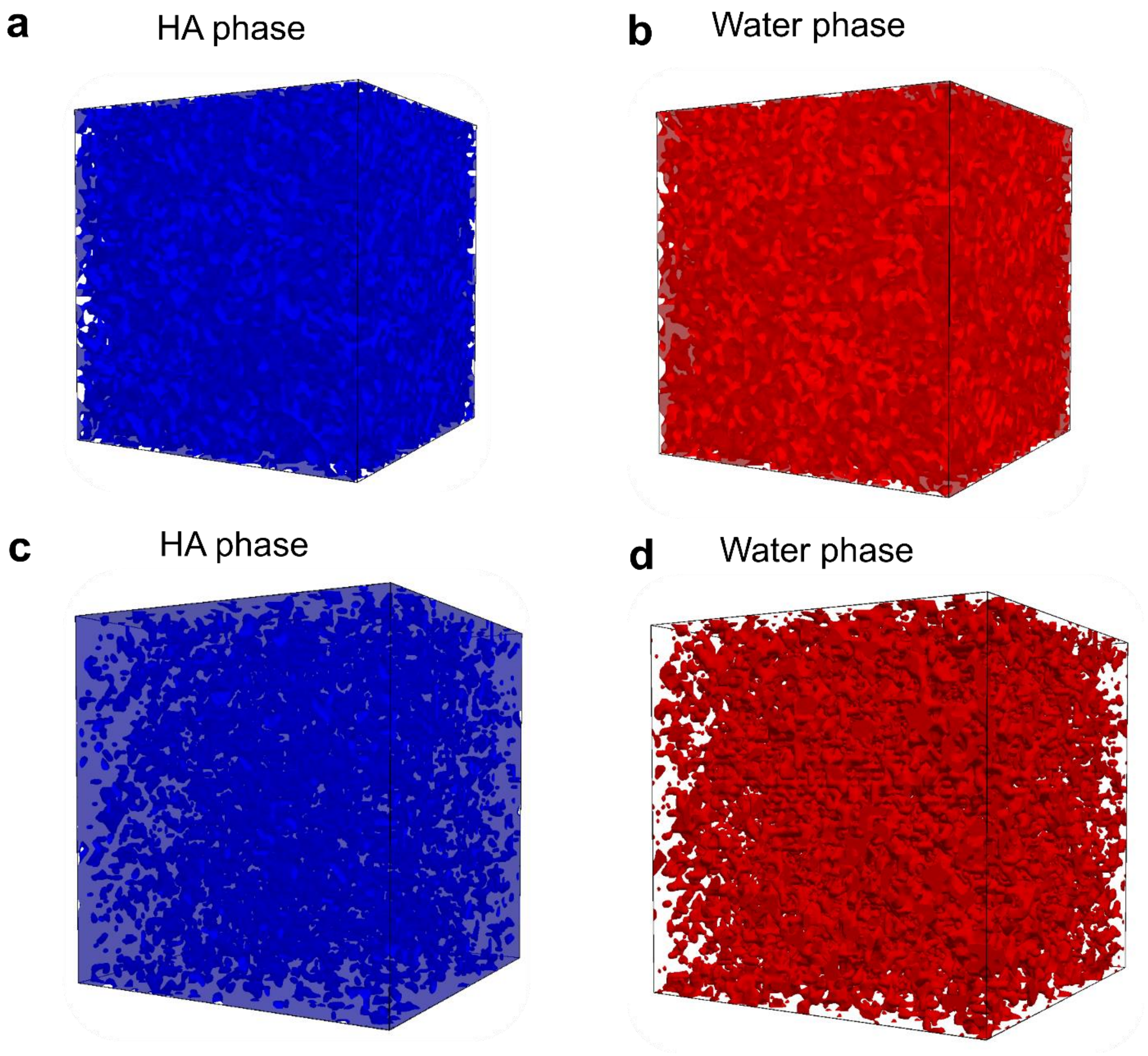

Figure S7. 3D representative morphologies generated using SAXSMorph showing HA phase (blue) and water phase (red) for D_2O/HA : 80/20 (a,b) and D_2O/HA : 20/80 (c,d) samples. The edge length of the cubes is 25 nm.

<u>Section S2: Sample D_2O:HA (95:5)</u>

The scattering profile of J-aggregates after addition of 5 volume % HA is a superposition of J-aggregates and D_2O/HA (95/5) blend solution (Figure S8). Compared to the pure samples, the J-aggregate scattering intensity was increased by orders of magnitude, but did not change its general shape. On the other side, the scattering contribution from the D_2O/HA solution decreased significantly. One must conclude that HA forms complexes with

the J-aggregate, an HA environment will increase the scattering contrast, while a D_2O environment will decrease it. Scattering intensity is proportional to the scattering contrast squared.

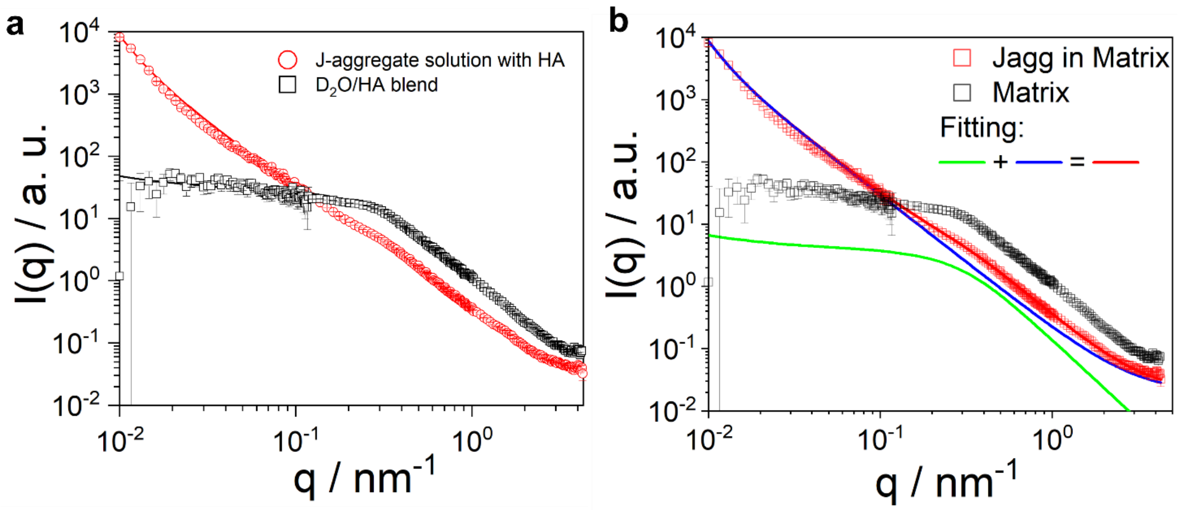

Figure S8. SANS scattering profiles of a 5 mM J-aggregate solution, where HA had been added to, compared to a D_2O/HA blend solution at the same volume fraction D_2O/HA : 95/05 (a). The J-aggregate spectrum is a superposition of the spectra of the D_2O/HA blend with much lower scattering intensity (green solid line) and an exponential background function with an exponent $\alpha = 2.15$ (blue solid line).

The scattering intensity profile from the D_2O/HA solution could be fitted best with a general gaussian coil as form factor. The particles interact strongly and a mass fractal with a Gaussian cut-off as structure factor was introduced.

The form factor is given by[3]:

$$P(q) = 2\int_0^1 dx(1-x)e^{-\frac{q^2R_g^2}{6}(2\upsilon+1)(2\upsilon+2)x^{2\upsilon}} \quad \text{(S3)}$$

whereby R_g is the radius of gyration and ν is the excluded volume parameter.

The structure factor is given as mass fractal with Gaussian cut-off[4]:

$$S(q) = 1 + \frac{D}{r_0^D}\int_0^\infty r^{D-3} h(r,\xi) \frac{\sin(qr)}{qr} r^2 dr \qquad (S4)$$

with

$$h(r,\xi) = exp\left[-\left(\frac{r}{\xi}\right)^2\right] \qquad (S5)$$

The fitting parameters to the data are given in Table S2. Both datasets were fitted simultaneously with the same set of parameters (besides I_0).

Table S2: Fitting parameters to form factor and structure factor for scattering functions of D_2O/HA (95/05) blends and a sample containing J-aggregates.

Sample	Form factor			Structure factor		
	R_g / nm	ν	I_0	r_0 / nm	ξ / nm	D
D_2O:HA	5.58	0.39	27.87	213.23	1111	1.06
J-agg in D_2O:HA	5.58	0.39	3.89	213.23	1111	1.06

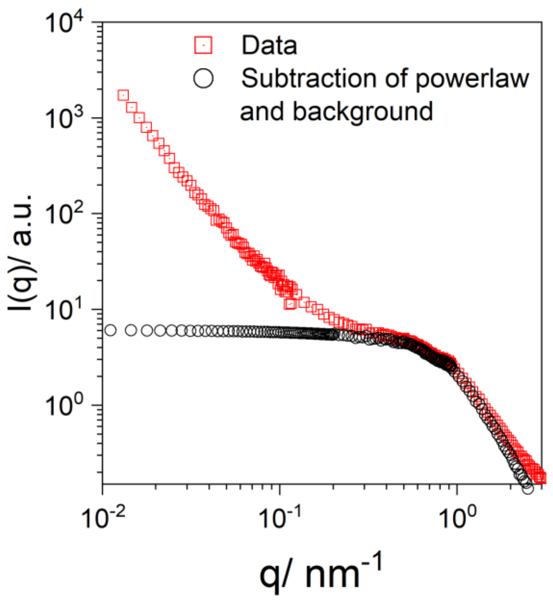

Figure S9. SANS scattering spectrum of the “emissive” J-aggregate (5 mM in D_2O after addition of HA summing to a volume ratio D_2O/HA = 80/20) and after subtraction of a powerlaw function with an exponent of 2.6 and background.

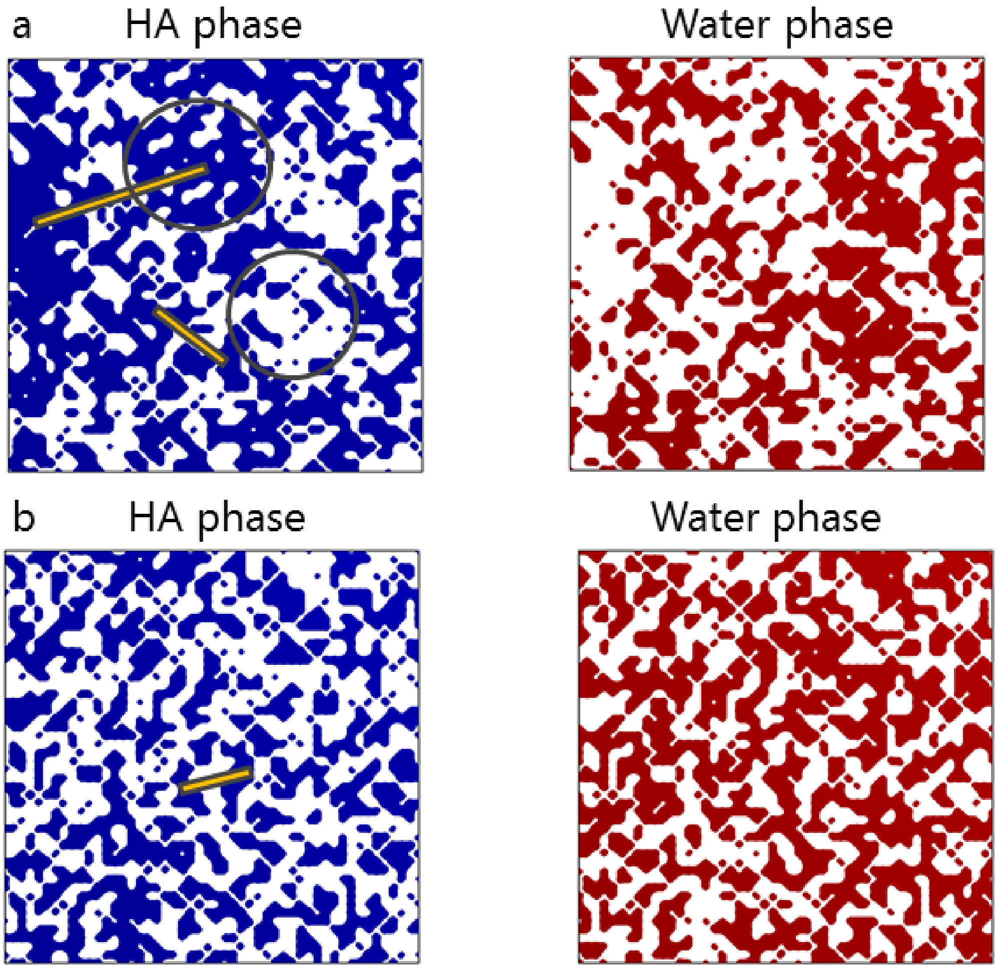

Figure S10. 2D slices of the morphologies shown in Figure 3d and 3f, main text. (a) 5 mM J-aggregate solutions in water after adding HA, (b) top phase of a 80/20 : D_2O/HA blend after having added dye to it. The circles exemplary indicate regions enriched in one phase, the bars represent the dominating length scale of the system following the SANS spectra. The edge length of the cubes is 25 nm.

Table S3: Scattering length density of D_2O, HA and TDBC

	**Scattering length density/ cm^{-2}**
D_2O	6.33×10^{10}
HA	-0.31×10^{10}
TDBC	1.64×10^{10}

Section S3: Variation in the amine water blend

In the main text we linked the morphology of the water/HA to the phase of the dye TDBC (emissive J-aggregate, non-emissive J-aggregate or monomer). The emission behavior was investigated in detail for the dye in a blend with EA and HA. Here we screen the aggregation behavior of the dye TDBC in a couple of additional water/amine blends. In the blends the different phases have a characteristic color appearance. An emissive J-aggregate is also formed with Propylamine and Octylamine, while non-emissive J-aggregates seem to dominate in Triethylamine and N-Methyldioctylamine (see Figure S11). The dye also shows a very different behavior in the amines alone. The insolubility of TDBC in Triethylamine and N-Methyldioctylamine confirms our claim that monomer solubility in the amine phase supports the formation of emissive J-aggregates. As shown in Figure S12, the difference between ethanolamine and ethylamine is the presence of -OH group and alkyl group, respectively. For 1:1 ratio of water/amine, there is a stark change in PLQY from 5% to 45%. This is due to reduced dissolution of monomers in ethanolamine compared to the alkyl group in ethylamine.

Table S4: Dye assembly in blends water and amines, and dye phase in the amine

Amine	H_2O/amine	Dye in blend	Dye in amine
Triethylamine (tertiary)	2-phase	Non-emissive J-aggregate	insoluble
N-Methyldioctylamine (secondary)	2-phase	Non-emissive J-aggregate	insoluble
Dimethylamine (secondary)	1-phase	Monomer	Monomer
Propylamine (primary)	1 phase	Emissive J-aggregate	Monomer/non-emissive J-aggregate/scattering
Octylamine (primary)	2-phase	Emissive J-aggregate	Monomer/non-emissive J-aggregate/scattering
Ethanolamine (primary amine and primary alcohol)	1-phase	Emissive J-aggregate	Monomer/emissive J-aggregate

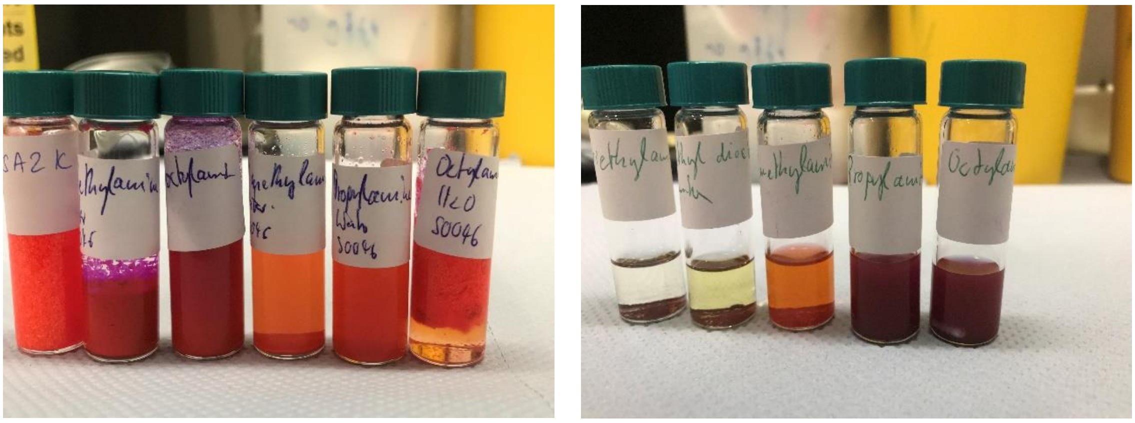

Figure S11. Photographs of the dye TDBC in blends of water with Ethylamine (reference), Triethylamine, N-Methyldioctylamine, Dimethylamine, Propylamine and Octylamine (left image, from left to right) and in the amine only (right image). From UV-Vis spectroscopy (data not shown here) we could identify a mixture of monomer and J-aggregate in the TDBC solutions in Propylamine and Octylamine.

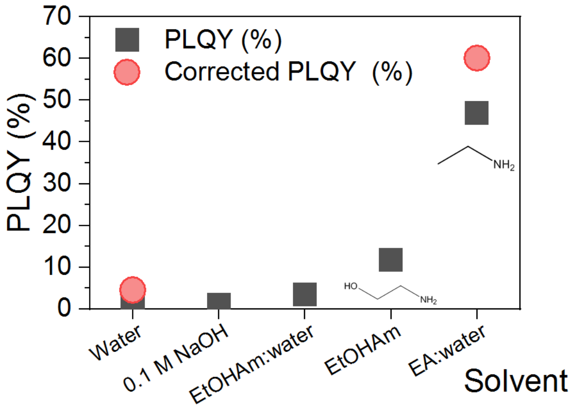

Figure S12. PLQY for TDBC in different solvents – water, 0.1 M NaOH, Ethanolamine-water (1:1, EtOHAm:Water), Ethanolamine (EtOHAm) and Ethylamine-water (1:1, EA:Water).

Section S4: Additional cyanine dyes

We screened a couple of cyanine dyes for their behavior in the HA/H_2O blend. Even though some dyes are structurally very similar to TDBC (Figure S13a), none of them formed J-aggregates in the HA/H_2O blend.

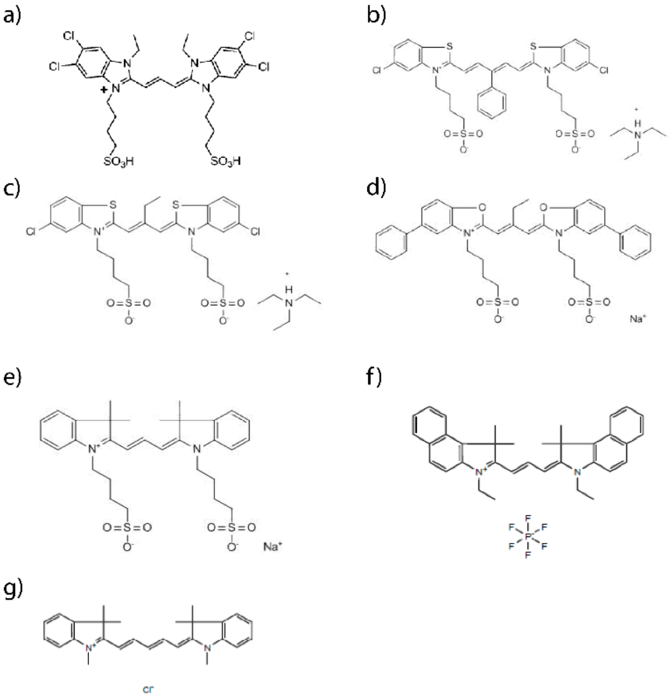

Dye	in HA / H_2O blend
**a)**	Emissive J-aggregate
**b)**	Degrades
**c)**	Monomer
**d)**	Degrades
**e)**	Monomer
**f)**	Monomer
**g)**	Monomer

Figure S13. Structures of cyanine dyes used in our study. a) TDBC used in the main study, b) 5-Chloro-2-[5-[5-chloro-3-(4-sulfobutyl)-3H-benzothiazol-2-ylidine]-3-phenyl-penta-1,3-dienyl]-3-(4-sulfobutyl)-benzothiazol-3-ium hydroxide, inner salt, triethylammonium salt (S2284), c) 5-Chloro-2-[2-[5-chloro-3-(4-sulfobutyl)-3H-benzothiazol-2-ylidinemethyl]-but-1-enyl-3-(4-sulfobutyl)-benzothiazol-3-ium hydroxide, inner salt, triethylammonium salt (S2278,THIATS), d) 5-Phenyl-2-[2-[[5-phenyl-3-(4-sulfobutyl)-3H-benzoxazol-2-ylidine]-but-1-enyl-3-(4-sulfobutyl)-benzoazolium hydroxide, inner salt, sodium salt (S0271), e) 2-[3-[3,3-Dimethyl-1-(4-sulfobutyl)-1,3-dihydro-indol-2-ylidine]-3,3-dimethyl-1-(4-sulfobutyl)-3H-indolium hydroxide, inner salt, sodium salt (S0522), f) 3-Ethyl-2-[3-(3-ethyl-1,1-dimethyl-1-3-dihydro-benzo[e]indol-2-ylidine)-propenyl]-1,1-dimethyl-1H-benzo[e]indolium hexafluorophosphate (S0666), g) 1,3,3-Trimethyl-2-[5-(1,3,3-trimethyl-1,3-dihydro-indol-2-

ylidine)-penta-1,3-dienyl]-3H-indolium chloride (S0944). The dyes shown in b), c) and d) are well known aggregate forming dyes. The table summarizes the dye phase from UV-Vis spectra. All dyes were purchased from FEW Chemicals, Germany.

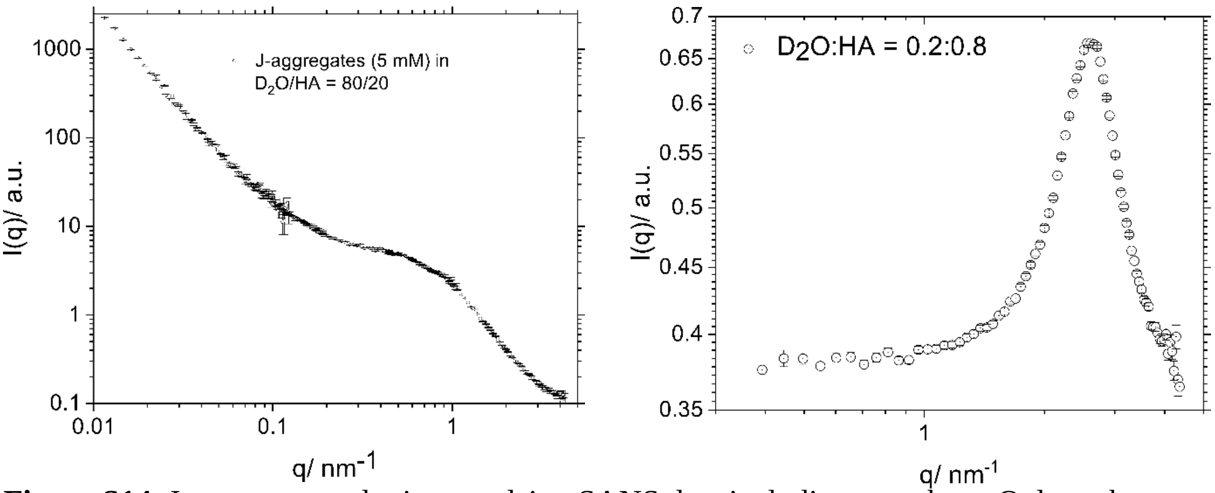

Figure S14. J-aggregate and microemulsion SANS data including error bars. Only at the extreme q-values for each detector setting the errors are visible in the graph.

References

[1] G. M. Akselrod, Y. R. Tischler, E. R. Young, D. G. Nocera, V. Bulovic, *Phys. Rev. B. Lumin.* **2010,** 82, 113106.

[2] M. Teubner, R. Strey, *J. Chem. Phys.* **1987**, *87*, 3195.

[3] B. Hammouda, in *Polym. Charact.*, Springer Berlin Heidelberg, Berlin, Heidelberg, **1993**, pp. 87–133.

[4] C. M. S. and G. M. Wang, *Phys. Rev. E* **1999**, *60*, 7143.
